# Comparison of capillary gas chromatographic method and automated spectrophotofluorometric methods for measuring content uniformity of conjugated oestrogens in pharmaceutical preparations

**DOI:** 10.1155/S1463924691000226

**Published:** 1991

**Authors:** J. D. Russell, J. Mumley, M. Spring, A. Kutz, R. N. Johnson

**Affiliations:** Analytical Research and Services Wyeth-Ayerst Research 64 Maple Street Rouses Point New York 12979 USA

## Abstract

To measure the content uniformity of conjugated cestrogens tablets, the USP XXII monograph specifies a capillary gas chromatographic method. This involves separation of the trimethylsilyl derivatives of the various oestrogens found in a typical conjugated oestrogens tablet using a fused-silica 0V 225 bonded phase column. Hydrogen is used as the carrier gas with FID detection. A more rapid spectrophotofluorometric method of analysis has been developed in which the oestrogens are extracted from the dosage form into water, then transferred into organic solvent as the dicyclohexylamine complex. Fluorescence is developed by heating the solution of the complex in the presence of 70% sulphuric acid. The chemistry and detection are carried out using a segmented-flow analyser.

The spectrophotofluorometric method is preferred as a routine control for content uniformity because considerable time is saved during sample preparation and analysis compared to gas chromatography. Coefficients of variation show that both methods produce acceptable results. These results fall well within the USP XXII [1] monograph, limits and also well within the tighter limits imposed by the USP XXII, section 905.


Journal of Automatic Chemistry, Vol. 13, No. 3 (May-June 1991), pp. 115-117

### Comparison of capillary gas chromatographic method and automated spectrophotofluorometric methods for measuring content uniformity of conjugated oestrogens in pharmaceutical preparations

**J. D. Russell, Jr, J. Mumley, M. Spring, A. Kutz and R. N. Johnson**
*Analytical Research and Services, Wyeth-Ayerst Research, 64 Maple Street, Rouses Point, New York, 12979, USA*

*To measure the content uniformity of conjugated oestrogens tablets, the USP XXII monograph specifies a capillary gas chromatographic method. This involves separation of the trimethylsilyl derivatives of the various oestrogens found in a typical conjugated oestrogens tablet using a fused-silica OV225 bonded phase column. Hydrogen is used as the carrier gas with FID detection. A more rapid spectrophotofluorometric method of analysis has been developed in which the oestrogens are extracted from the dosage form into water, then transferred into organic solvent as the dicyclohexylamine complex. Fluorescence is developed by heating the solution of the complex in the presence of 70% sulphuric acid. The chemistry and detection are carried out using a segmented-flow analyser.*

*The spectrophotofluorometric method is preferred as a routine control for content uniformity because considerable time is saved during sample preparation and analysis compared to gas chromatography. Coefficients of variation show that both methods produce acceptable results. These results fall well within the USP XXII [1] monograph, limits and also well within the tighter limits imposed by the USP XXII, section 905.*

### Introduction

Pharmaceutical preparations containing conjugated oestrogens are formulated with either synthetic or naturally derived oestrogens. The conjugated oestrogens used in this study were isolated from pregnant mare's urine. These conjugated oestrogens consist of a mixture of ten oestrogens as the sodium sulphated salts. The major components of this mixture are sodium oestrone sulphate, sodium equilin sulphate, sodium 17-alpha dihydroequilin sulphate, sodium 17-alpha-oestradiol sulphate, sodium 17-beta-dihydroequilin sulphate and sodium delta-8,9-dihydroestrone sulphate [2]. Conjugated oestrogens are used in the treatment of hormonal deficiences. To ensure that the final pharmaceutical preparation has the composition required to be therapeutically effective, it is essential that rigorous analytical testing be performed. A highly specific gas chromatographic procedure is currently used for testing strength, composition and content uniformity. This method, however, is not efficient for testing the large number of individual units required to demonstrate uniformity.

An automated spectrofluorometric method based on segmented continuous-flow technology has been developed.

Data are presented to demonstrate that this alternate method produces results that are equivalent to the gas chromatographic method, offering advantages in terms of speed and throughput.

### Materials and methods

The conjugated oestrogens tablets used in this study were obtained from commercial batches of Premarin [3].

### *Gas chromatographic method*

Details of the gas chromatographic method were presented in the USP [1] monograph for *Conjugated Estrogen Tablets*.

### *Automated fluorescence method*

The procedure is based on the development of fluorescent chromophores produced by an extract of conjugated oestrogens with 70% sulphuric acid. The chemistry is automated using a segmented-flow system. The method consists of the following steps:

(1) The conjugated oestrogens are extracted from each tablet with water.
(2) The conjugated oestrogens are extracted into organic solvent as the dicyclohexylamine complex.
(3) The dicyclohexylamine complex is extracted into hot sulphuric acid to develop fluorescence.
(4) Quantitation is achieved by comparison to similarly tested aliquots of a reference pool to which a strength has been assigned by gas chromatographic assay.

Details of the flow rates used for the method are shown in figure 1.

### *Equipment*

AutoAnalyzer® modules, available from Technicon, Inc., Tarrytown, New York (or equivalent), consisting of a sampler, proportioning pump, heating bath, fluorometer and recorder.

0142-0453/91 $3.00 © 1991 Taylor & Francis Ltd.

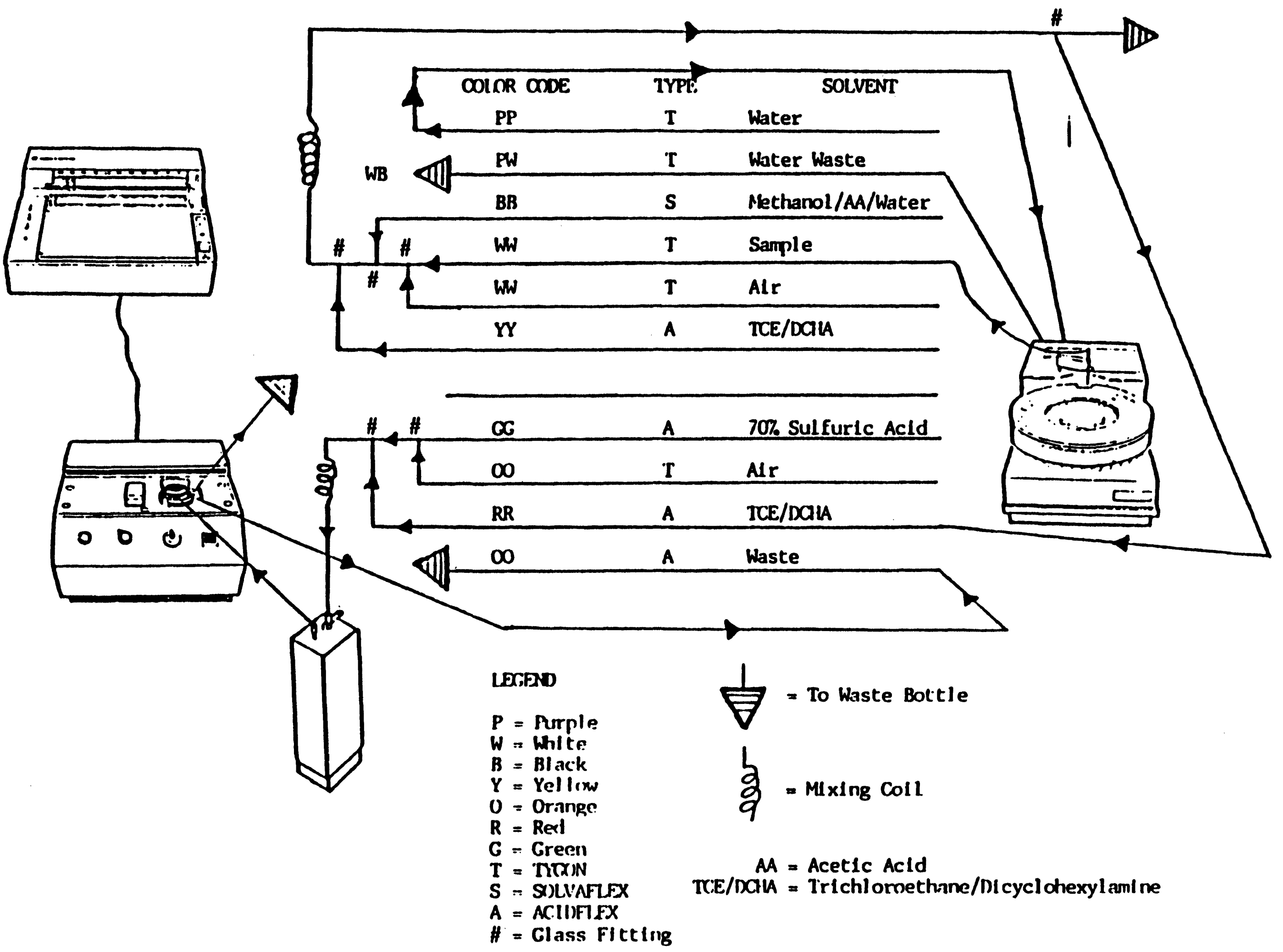

*Figure 1. Flow diagram and equipment set-up for automated method.*

*Reagents*

The following reagents are used: dicyclohexylamine acetate – melting range 114 °C to 118 °C as determined by the USP class 1a procedure; sulphuric acid, 70% solution; acetic acid : methanol : water solution – 1 : 1 : 2; dicyclohexylamine acetate solution 0·15% in 1,1,2-trichloroethane.

*Equipment preparation*

The AutoAnalyzer modules are connected as shown in figure 1 and the heating bath adjusted to 95–100 °C. The fluorometric detector has an excitation wavelength at 415 nm and an emission wavelength at 525 nm. Reagents are pumped through the system until a stable base-line is produced. The sampling rate and sample to wash ratio are adjusted, so that well-formed, reproducible peaks are obtained (see figure 2). A sampling rate of 20 samples per hour and a sample to wash ratio of 1 : 3·5 are typical conditions.

*Preparation of the reference tablet pool*

Ten or more tablets are taken and the dye and sugar coats removed by rinsing with water. The tablets are transferred to a container and sufficient water added to

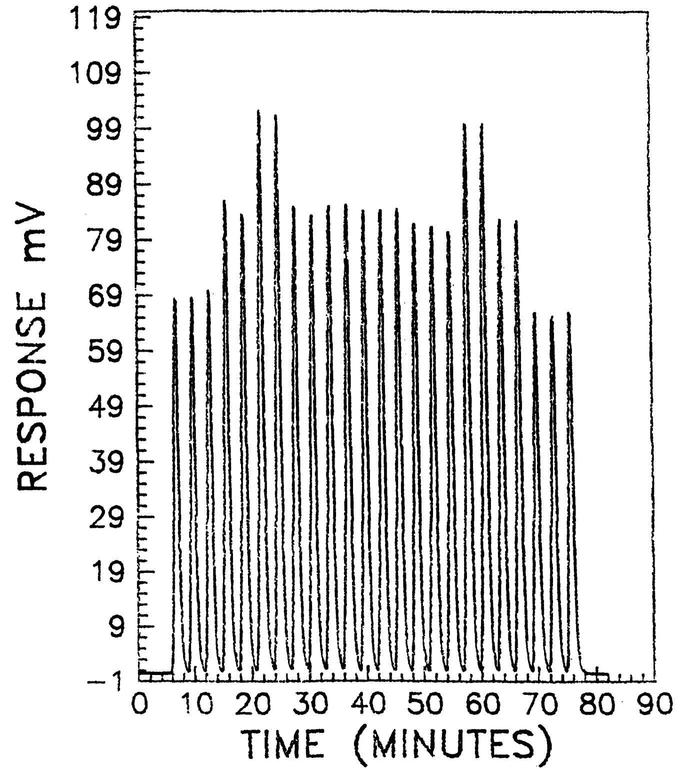

*Figure 2. Conjugated oestrogens autoanalyser trace.*

*Table 1. Comparison of data obtained during routine testing of Premarin tablets using the official gas chromatographic method and the automated procedure.*

| | | For sum of two oestrogens* Automated method | | Gas chromatography | | For sum of three oestrogens** Automated method | | Gas chromatography | |
|---|---|---|---|---|---|---|---|---|---|
| Batch | Claim mg/tab | % Claim found | CV(%) | % Claim found | CV(%) | % Claim found | CV(%) | % Claim found | CV(%) |
| A | 0·900 | 87·4 | 2·85 | 87·1 | 2·97 | 103·0 | 2·86 | 103·0 | 2·87 |
| B | 0·900 | 86·9 | 1·79 | 84·1 | 1·40 | 104·4 | 1·79 | 101·0 | 1·30 |
| C | 2·500 | 82·4 | 3·00 | 82·8 | 2·60 | 98·8 | 3·00 | 99·6 | 3·10 |
| D | 1·250 | 84·8 | 2·75 | 81·6 | 3·31 | 101·6 | 2·75 | 98·4 | 3·09 |
| E | 1·250 | 84·8 | 1·93 | 81·6 | 3·07 | 101·6 | 1·93 | 97·6 | 2·69 |
| F | 1·250 | 88·0 | 3·41 | 85·6 | 2·78 | 104·8 | 3·40 | 101·6 | 2·82 |
| G | 0·625 | 86·1 | 2·40 | 86·9 | 2·13 | 102·9 | 2·40 | 104·3 | 2·13 |
| H | 0·625 | 90·1 | 3·29 | 80·6 | 5·74 | 107·7 | 3·28 | 97·8 | 5·16 |
| I | 0·625 | 85·9 | 1·27 | 87·5 | 4·89 | 101·8 | 1·20 | 103·7 | 4·82 |
| J | 0·625 | 84·8 | 2·97 | 86·6 | 3·01 | 101·9 | 2·98 | 103·5 | 2·85 |
| K | 0·625 | 93·6 | 2·54 | 84·5 | 2·00 | 111·8 | 2·56 | 101·9 | 2·08 |
| L | 0·625 | 87·0 | 3·15 | 86·2 | 1·42 | 102·9 | 3·15 | 102·1 | 1·42 |
| M | 0·300 | 90·3 | 2·20 | 90·3 | 1·36 | 107·0 | 2·20 | 108·3 | 1·34 |
| N | 0·300 | 91·0 | 2·49 | 83·0 | 1·87 | 108·7 | 2·47 | 99·3 | 2·01 |
| | Means | 87·4 | 2·57 | 84·9 | 2·75 | 104·2 | 2·57 | 101·6 | 2·69 |
| | SD | 3·0 | — | 2·8 | — | 3·5 | — | 3·0 | — |
| | CV(%) | 3·4 | — | 3·3 | — | 3·3 | — | 2·9 | — |

* For the sum of two oestrogen determinations, sodium estrone sulphate and sodium equilin sulphate are measured and account for about 85% of the total claim.

** For the sum of three oestrogen determinations, sodium estrone sulphate and sodium equilin sulphate and sodium 17-alpha-dihydroequilin sulphate are measured and account for 100% of the claim.

produce a final extract (based on product strength) of between 0·12 and 0·25 mg/ml. The tablets are mixed vigorously until dissolution is complete. From the resultant extract 8, 10, and 12 ml are diluted to 100 ml with water. Reference extracts of about 80%, 100%, and 120% of the expected concentration resulting from the individual sample extracts are thus produced.

*Preparation of samples*

The dye and sugar coat is removed from each tablet. Each tablet is placed in a container and sufficient water added to the container to produce a final extract (based on product strength) of between 0·012 and 0·025 mg/ml. The solution is vigorously mixed until dissolution of the tablet is complete.

*Procedure*

The individual tablet extracts are bracketed with the reference extracts and the automated analysis is performed. A least square linear regression is calculated from the reference responses ($y$) on reference concentration ($x$). The reference concentration is assigned based on the gas chromatographic potency of the batch being tested. The concentration of each sample preparation is obtained from the regression line.

### Results

For each batch, 10 individual determinations were normally conducted. The CV of the manual gas chromatographic method was shown to be 2·69–2·75%, while the CV of the automated fluorescent method was 2·57%, as shown in table 1. The USP method measures sodium estrone sulphate and sodium equilin sulphate. This is referred to as the 'sum of two oestrogens'. When sodium 17α-dihydroequilin sulphate is included in the potency, this is the 'sum of three oestrogens'.

For the 'sum of two oestrogens' the average % claim for the fluorescent and GC method were 87·4 and 84·9% respectively. Results for the 'sum of three oestrogens' were 104·2 and 101·6% respectively. Thus, the results of testing 14 batches of Premarin by both methods (see table 1) indicate that the means are comparable.

The coefficient of variation between batches is 2·9–3·4%. Both methods show that the batches are very consistent in quality.

### Conclusion

The automated method is suitable for use as a routine control testing procedure and has, in fact, received the approval of the Food and Drug Administration. Implementation of the method has reduced the testing time for content uniformity from 16 to 3 hours per batch without compromising quality.

### References

1. The United States Pharmacopeial Convention, Inc., *The United States Pharmacopeia,* XXII ed., including *Supplements* (Mack Printing Co., Easton, Pennsylvania, 1990.
2. Johnson, R. and Lyman, G., *Journal of Chromatography,* **234** (1982), 234.
3. Trademark of Wyeth-Ayerst Laboratories, Division of American Home Products Corporation.

